# The cost-effectiveness of pegaspargase versus native asparaginase for first-line treatment of acute lymphoblastic leukaemia: a UK-based cost-utility analysis

**DOI:** 10.1186/s13561-019-0257-3

**Published:** 2019-12-29

**Authors:** Xingdi Hu, Kingsley P. Wildman, Subham Basu, Peggy L. Lin, Clare Rowntree, Vaskar Saha

**Affiliations:** 1 GHEOR Analytics, Shire, Cambridge, MA USA; 2 Medical Affairs for Oncology, UK & Ireland, Servier Laboratories Ltd, Stoke Poges, UK; 3 Medical Affairs Oncology, UK & Republic of Ireland, Shire Pharmaceuticals Ltd, London, UK; 4 Haematology, University Hospital of Wales, Cardiff, UK; 5 Division of Cancer Sciences, School of Medical Sciences, Faculty of Biology, Medicine and Health, University of Manchester, The Oglesby Cancer Research Building, 555 Wilmslow Road, Manchester, M20 4GJ UK; 6 Tata Translational Cancer Research Centre, Tata Medical Center, Kolkata, India

**Keywords:** Acute lymphoblastic leukaemia, Asparaginase, Cost-effectiveness, First line treatment

## Abstract

**Background:**

L-asparaginase is a key component of treatment for patients with acute lymphoblastic leukaemia (ALL) in the UK. Commonly used forms of asparaginase are native *E. coli*-derived asparaginase (native asparaginase) and pegaspargase in first-line combination therapy, and native *Erwinia chrysanthemi*-derived asparaginase (Erwinia asparaginase) as second-line treatment. The objective of this study was to evaluate the cost-effectiveness of pegaspargase versus native asparaginase in first-line combination therapy for patients with newly diagnosed ALL. A combined decision tree and health-state transition Markov cost-effectiveness model was developed to assess the relative costs and health outcomes of pegaspargase versus native asparaginase in the UK setting.

**Results:**

In base case analyses, first-line pegaspargase (followed by Erwinia asparaginase in cases of hypersensitivity) dominated first-line native asparaginase followed by Erwinia asparaginase; i.e. resulted in lower costs and more quality-adjusted life year gain. The favourable hypersensitivity rates and administration profile of pegaspargase led to lifetime cost savings of £4741 versus native asparaginase. Pegaspargase remained cost-effective versus all treatment strategies in all scenario analyses, including use of the 2500 IU/m^2^ dose, recommended for patients ≤21 years of age.

**Conclusions:**

Pegaspargase, as part of multi-drug chemotherapy, is a cost-effective option for the treatment of newly diagnosed ALL. Based on this study, The National Institute for Health and Care Excellence Technology Appraisal Committee concluded that it could recommend pegaspargase as a cost-effective use of National Health Service resources in England & Wales for treating ALL in children, young people and adults with untreated, newly diagnosed disease.

**Trial registration:**

UKALL 2011, EudraCT number 2010-020924-22; UKALL 2003, EudraCT number 2007-004013-34; UKALL14, EudraCT number 2009-012717-22.

## Background

Acute lymphoblastic leukaemia (ALL) is an acute, rapidly progressing, and life-threatening cancer that accounts for less than 1% of all new cancer diagnoses in the United Kingdom (UK) [1]. The incidence of ALL is highest in children (0–4 years old) and lowest in adults aged 30 to 39 years; 6% of new cases were in adults aged ≥75 years in the UK in 2016 [2]. Males have higher incidence rates than females across most UK age groups [2].

As outlined by national protocols, the treatment for patients with Philadelphia chromosome-negative ALL in the UK involves the administration of combination chemotherapy, including L-asparaginase as an essential component, and the majority of adults progress to an allograft following successful induction therapy [3–8]. Asparaginase is an enzyme derived from bacteria that deaminates circulating asparagine and glutamine, thereby negatively affecting ALL blasts, which are unable to synthesise asparagine [9]. Three forms of asparaginase are commonly used in clinical practice: native *Escherichia (E.) coli*-derived asparaginase (native asparaginase), native *Erwinia chrysanthemi*-derived asparaginase (Erwinia asparaginase) and pegaspargase, an *E. coli*-derived asparaginase modified by pegylation (conjugation to polyethylene glycol). The frequency and methods of administration, approved line of treatment and rates of hypersensitivity differ among these three treatment options, all of which impacts on clinical management of patients with ALL.

Historically, native asparaginase was the first-line standard of care asparaginase in Europe and the United States. It is highly immunogenic, with increased antibody levels observed in 26% of patients receiving native asparaginase versus 2% of patients receiving pegaspargase in a randomised study [10, 11]. Native asparaginase also results in higher levels of hypersensitivity, with an incidence of ≤76% any-grade and ≤ 15% grade 3 versus 1–30% any grade and 2–5% grade 3 reactions with pegaspargase [12]. In addition, pegaspargase has a longer half-life (5.5–7 days for pegaspargase versus 8–30 h [native asparaginase] and 5.9–16 h [Erwinia asparaginase]), requiring less frequent dosing, and is associated with similar long-term outcomes (event-free survival [EFS] and overall survival [OS]) to other asparaginases [10, 13–16]. Patients who develop significant hypersensitivity (grade ≥ 3) to first-line native asparaginase or pegaspargase, or who have low asparaginase activity levels, should be switched to Erwinia asparaginase [13].

In the UK, paediatric, adolescent and young adult (AYA) patients up to 25 years of age with newly diagnosed ALL are treated on the current national trial, UKALL 2011 (EudraCT number 2010–020924-22), successor to UKALL 2003 (EudraCT number 2007–004013-34), which had a similar chemotherapy backbone [6, 17–19]. Adults with newly diagnosed ALL aged 25–59 years, and those aged 60–65 years with no comorbidities, are treated on the UKALL14 trial (EudraCT number 2009–012717-22) [3, 7]. Favourable outcomes and tolerability with 1000 IU/m^2^ pegaspargase, used in more than 97% of the eligible paediatric ALL population in UKALL 2003 [17, 18], led to the incorporation of pegaspargase at this dose in UKALL 2011 and this is also the dose used in UKALL14. In adults, the UK regimen and dose was further modified in patients > 40 years of age (one pegaspargase induction dose removed) and those with Philadelphia-positive ALL (no pegaspargase given) following a high level of adverse events during induction [20].

A number of studies in the USA and the Netherlands have reported similar costs or cost savings with pegaspargase versus Erwinia and/or native asparaginase treatment [12, 21–24]. However, prior to our study, no direct comparisons had been performed on the cost-effectiveness of pegaspargase, native asparaginase, and Erwinia asparaginase as the key component of multi-agent chemotherapy for treating ALL. Our analysis aimed to fill this gap and formed part of a formal National Institute for Health and Care Excellence (NICE) technology appraisal of pegaspargase in the UK [25]. As pegaspargase is approved as first-line treatment, the objective of this study was to evaluate the cost-effectiveness of pegaspargase compared with native asparaginase, another possible first-line asparaginase option, as part of antineoplastic combination therapy for treating patients with newly diagnosed ALL in the UK.

## Methods

### Model overview

A cost-effectiveness model was developed to assess the relative costs and health outcomes of pegaspargase versus native asparaginase as part of first-line combination chemotherapy for patients with ALL who are treated on UK national protocols. The reporting of this economic evaluation utilised the ISPOR guideline Consolidated Health Economic Evaluation Reporting Standards checklist (Additional file 1) [26].

The cost-effectiveness model comprised a decision-tree (DT) and a state-transition Markov model. The DT model included treatment regimen cycles, occurrence of hypersensitivity to asparaginase and asparaginase switch. A 5-year time horizon was used for the DT model because first-line chemotherapy for ALL, including maintenance treatment, is usually completed within 3.5 years. As patients experience a survival outcome during and after treatment, a Markov model was also incorporated in parallel to extrapolate over a life-time horizon.

The analysis was conducted from the perspective of the UK National Health Service (NHS). Direct medical costs were estimated for asparaginase acquisition and their administration. Out-of-pocket costs and indirect costs, such as productivity loss, were excluded. Costs were expressed in GBP (£; year 2016). Health outcomes were measured as quality-adjusted life years (QALYs), which is the preferred indicator of effectiveness for NICE. Both costs and health outcomes were discounted at 3.5% per annum, in line with the NICE guidelines [25].

Cost-effectiveness was determined by the incremental cost-effectiveness ratio (ICER), measured as incremental costs per QALY gained. Deterministic and probabilistic sensitivity analyses (Monte Carlo simulation) were performed to assess the robustness of the model results. The model was developed using Microsoft Excel 2010 and Visual Basic Applications.

### Model structure

Cost effectiveness was assessed using a 5-year DT model and a lifetime Markov model in line with published UK ALL protocols and their findings, where available.

#### DT model

**Fig. 1:**
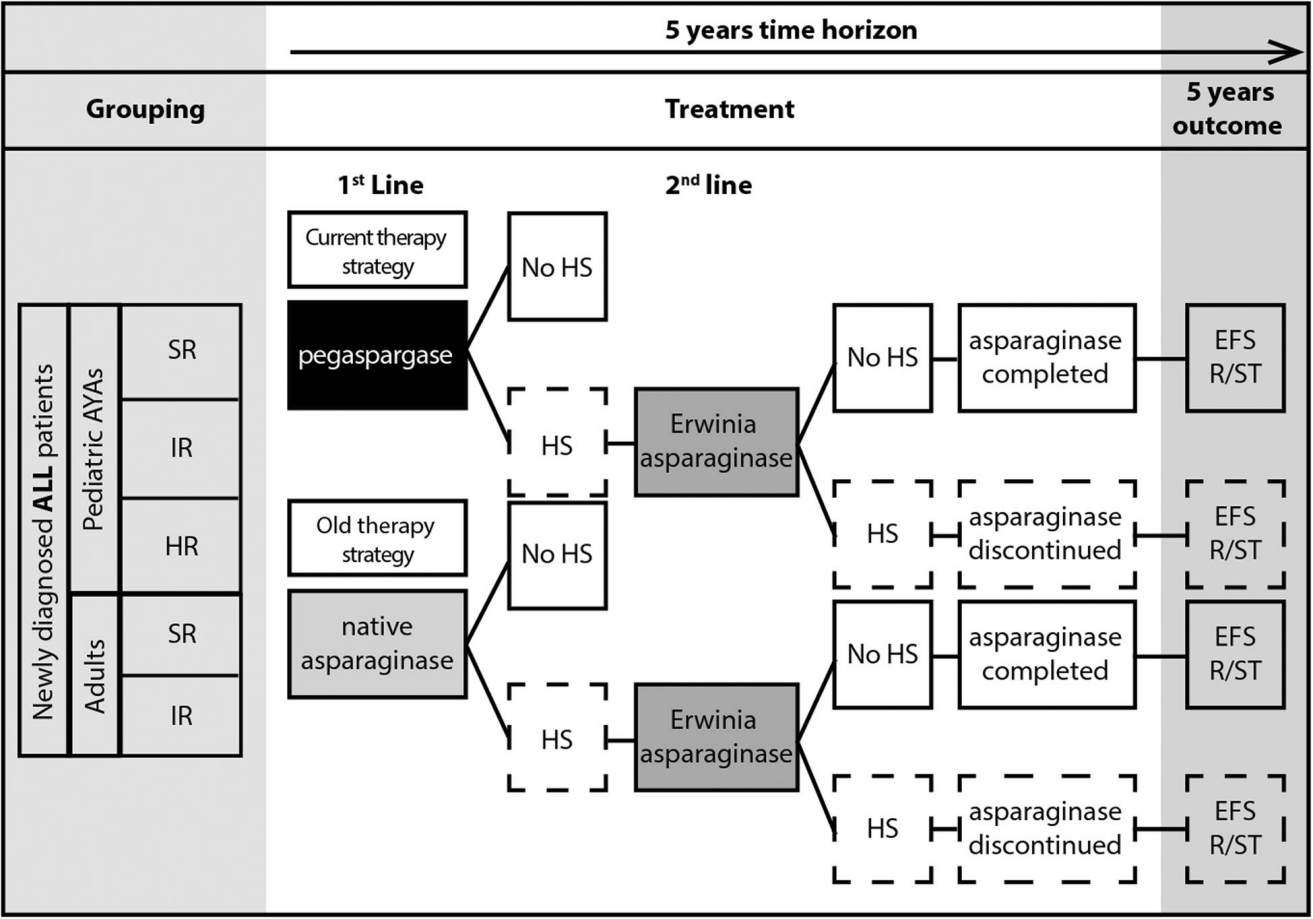
Overview of asparaginase treatment scenarios in the decision-tree model. Two scenarios were modelled, according to potential clinical options for first-line and second-line (in case of hypersensitivity) asparaginase treatment in the UK. Current therapy strategy: pegaspargase followed by Erwinia asparaginase. Old therapy strategy: native asparaginase followed by Erwinia asparaginase. ALL, acute lymphoblastic lymphoma; AYA, adolescents and young adults; EFS, event-free survival; HR, high risk; HS, hypersensitivity; IR, intermediate risk; R/ST, relapse/secondary tumor; SR, standard risk; y, year

The DT model allows explicit modelling of patient flow according to treatment dosing, frequency and switching in case of asparaginase hypersensitivity, consistent with the UKALL 2003 protocol (paediatrics and AYA) and UKALL14 protocol (adults) (Fig. 1). The first-line standard of care treatment pathway in the UK is pegaspargase, followed by Erwinia asparaginase in cases of hypersensitivity (referred to hereafter as ‘current therapy strategy’) [3, 4]. Three alternative switching scenarios were requested by NICE: native asparaginase to Erwinia asparaginase (referred to hereafter as ‘old therapy strategy’; native asparaginase is no longer widely available), Erwinia asparaginase to pegaspargase and Erwinia asparaginase to native asparaginase. Of these, only the old therapy strategy was modelled and included in this manuscript, based on UK clinical practice. The other two scenarios, Erwinia asparaginase to pegaspargase and Erwinia asparaginase to native asparaginase, were not presented here as they do not align with approved indications and are not part of UK clinical practice. Furthermore, patients would not be switched between native asparaginase and pegaspargase because of the risk of cross reactivity or hypersensitivity due to both being derived from *E. coli*.

Six Erwinia asparaginase doses correspond to one pegaspargase dose if a hypersensitivity reaction occurs. As the decision problem was to compare the different types of asparaginase, concomitant medications were assumed to remain unchanged and were thus not assessed in this analysis. As such, the only differences between the two scenarios being compared (current versus old therapy strategy) lay in the sequencing, dosing, frequency, and cost of the asparaginase formulations, and the occurrence of hypersensitivity.

To accurately reflect therapy regimens during the treatment period, the DT model used cycles corresponding to different treatment stages of the UKALL 2003 and UKALL14 protocols, expressed in weeks. For paediatric and AYA patients, this corresponded to seven cycles across five treatment stages (induction, consolidation, interim maintenance, delayed intensification, and maintenance). For clinically high-risk (HR) patients, asparaginase was administered in all cycles listed above, except during maintenance. For standard (SR)-and intermediate-risk (IR) patients, asparaginase was administered during induction and delayed intensification. For adult patients, the model included five cycles across four treatment stages (induction, intensification, consolidation, and maintenance). Patients received asparaginase during induction, intensification and consolidation, but not maintenance. Patients were switched to a second-line asparaginase if hypersensitivity occurred and discontinued asparaginase if hypersensitivity occurred again.

#### Markov model

The Markov model began from ALL treatment initiation and ran in parallel with the DT model to account for the occurrence of relapse/secondary tumour and death during the treatment course. In paediatric and AYA patients aged ≤25 years old, the Markov model had 3 health states: EFS, survival with relapse/secondary tumour (R/ST), and death. In adult patients (aged from 26 to 65 years, treated according to the ongoing UKALL14 protocol), the Markov model had 2 health states: survival or death, based on the assumption that EFS and OS were equivalent for adult patients. For both paediatric and adult patients, only patients in the EFS state received asparaginase treatment per protocol. The Markov model continued to extrapolate patients’ survival beyond the end of chemotherapy.

#### Model inputs

The majority of inputs were obtained from a project-specific literature review. Table 1 outlines a summary of the inputs and their evidence sources. Clinical expert advice was utilised when relevant UK data were not available from the literature at the time of the study.

**Table 1:** Summary of input values in the model

Variable	Paediatric and AYA 74.4%			Reference (27)	Variable	Adults 25.6%		Reference (27)
Grouping (%)	HR	IR	SR	[18]	Grouping (%)	SR	HR	[3]
	21.4	29.1	49.5			32.1	67.9	
Age (years)	5	5	5	[18]	Age (years)	31	53	[1]
Body surface (m^2^)	0.75	0.75	0.75	RCPCH	Body surface (m^2^)	1.79	1.79	[33]
					Eligible for transplant (%)	47	47	[34]
Health utilities decrements (%)				Assumption (decrement from [28])	Health utilities decrements (%)			Assumption (decrement from [28])
Ind	25	25	25		Ind	25	25	
C	16	16	16		Int	25	25	
IM1	12	12	12		C1	12	12	
DI1	12	12	12		C3	12	12	
IM2	12	12	12		M	7	7	
DI2	12	12	12					
CT	7	7	7					
Hypersensitivity risk (%)					Hypersensitivity risk (%)			
Pegaspargase					Pegaspargase			
first line (Ind)	2	2	2	[18]	first line (Ind/Int)	2	2	Assumption
second line (DI/C)	2	2	2	[18]	second line (Int)	2	2	Assumption
native asparaginase					native asparaginase			
first line (Ind)	20	20	20	[29]	first line (Ind)	20	20	Assumption
second line (DI/C)	20	20	20	Assumption	second line (Int)	20	20	Assumption
Erwinia asparaginase					Erwinia asparaginase			
second line (DI/C)	37	37	37	[30]	second line (Int)	37	37	Assumption
Impact					Impact			
Utility decrement	0.014	0.014	0.014	[31]	Utility decrement	0.014	0.014	[31]
Cost (£)	470	470	470	[32]	Cost (£)	470	470	[32]
5-year outcomes					5-year outcomes			
Asparaginase completed					Asparaginase completed			
OS (%)	95	90	80	Assumption from [18]	OS (%)	40	30	[3]
EFS (%)	90	85	75		EFS (%)	–	–	Assumption
Asparaginase discontinued					Asparaginase discontinued			EFS=OS
RR for OS	0.95	0.95	0.95	Assumption	RR for OS	0.95	0.95	Assumption
RR for EFS	0.95	0.95	0.95	Assumption	RR for EFS	–	–	Assumption
								EFS=OS
Impact of R/ST^a^								
Utility decrement (%)^b^	20	20	20	Assumption				
Increased mortality (%)^c^	90	90	90	Assumption				

*C* Consolidation, *CT* Continuation, *DI* Delayed intensification, *EFS* Event-free survival, *HR* High risk, *IR* Intermediate risk, *IM* Interim maintenance, *Ind.* Induction, *Int.* Intensification, *M* Maintenance, *OS* Overall survival, *RCPCH* Royal College of Paediatrics and Child Health, *RR* Relative risk, i.e. EFS (asparaginase discontinued) = RR EFS (asparaginase completed), *R/ST* Relapse/Secondary Tumour, *SR* Standard risk

^a^Paediatric only. In Adults, EFS is assumed to equal OS

^b^Decrement (%) from EQ-5D age-specific UK population norms.

^c^2011–2013 data from England Life Tables [35].

### Survival for paediatric patients

For paediatric patients, the 5-year EFS and OS estimates for those who completed asparaginase treatment were derived from the UKALL 2003 trial results [17, 18]. In the base case, 5-year OS was 95, 90 and 80% for SR, IR and HR groups, respectively, and 5-year EFS was 90, 85 and 75% for the SR, IR and HR groups, respectively. For patients who discontinued asparaginase because of hypersensitivity to first- and second-line asparaginase, a reduction of 5% was assumed for EFS and OS (i.e. EFS when asparaginase discontinued = 0.95 x EFS when asparaginase completed).

When extrapolating to a lifetime horizon, beyond the 5 years modelled in the DT, EFS patients were considered as cured (validated as a reasonable assumption by expert opinion). These patients were subject to a general population mortality risk, taken from the Office of National Statistics life table for England, weighted by the male/female population from the UKALL 2003 study, i.e. 57% male and 43% female [17, 18, 35]. No further transition to R/ST was allowed in the model. R/ST patients were subject to an increased mortality risk (i.e. × 1.9 general mortality; validated as a reasonable assumption by expert opinion) and could no longer transition to the EFS health state.

### Survival for adult patients

For adult patients, a Weibull curve was fitted on 2 points: the assumed 5-year OS from the UKALL14 protocol (assuming 40% for patients ≤40 years and 30% for patients ≥41 years) and the 40-year OS set at 0% (i.e. all adult patients would have died 40 years after ALL diagnosis and treatment initiation). Similar to paediatric and AYA patients, adults who discontinued asparaginase because of hypersensitivity had an OS reduction of 5% applied.

### Risk of hypersensitivity

For pegaspargase, the risk of hypersensitivity leading to a treatment switch was assumed to be 2% for both first- and second-line asparaginase therapy, based on first-line hypersensitivity observed in UKALL 2003 [17, 18]; second-line hypersensitivity data were not reported. UKALL 2003 was considered to be the most appropriate study on which to base this assumption, given that it accounted for the majority of pegaspargase treatment in the UK at 1000 IU/m^2^ and the rates were validated by experts. The risk of hypersensitivity was assumed to be 20% for both first- [29] and second-line native asparaginase, and 37% for second-line Erwinia asparaginase [30].

The risk of hypersensitivity was assumed to be the same for both paediatric, AYA and adult patients, as validated by expert opinion. An adjustment was applied for the doses of native asparaginase and Erwinia asparaginase received in cases of hypersensitivity, because affected patients did not receive all doses of native asparaginase and Erwinia asparaginase for the corresponding treatment phase and hypersensitivity was likely to occur at the second injection, as indicated by expert opinion.

### Dose

In the base-case analysis, a 1000 IU/m^2^ dose was used for pegaspargase, per established UK protocols and clinical practice. In the scenario analyses, the summary of product characteristics (SmPC) dose of 2500 IU/m^2^ for patients ≤21 years of age was examined [13]. The analysed dose for native asparaginase was 10,000 IU/m^2^. The recommended dose of native asparaginase is 6000 IU/m^2^, three times per week [15], although a range of doses has been used historically, including 6000 IU/m^2^ twice weekly, 10,000 IU/m^2^ three times per week and 25,000 IU/m^2^ weekly [36]. The Erwinia asparaginase dose analysed (20,000 IU/m^2^) reflected use in the UKALL2003, UKALL2011 and UKALL14 protocols (6 × 20,000 IU/m^2^ doses to substitute for each dose of pegaspargase) [3–5], which is lower than the SmPC recommended dose of 25,000 IU/m^2^ [16].

### Transplant

In adult patients, sibling allogeneic transplant is currently the treatment of choice for eligible adults in first complete remission, according to the UKALL14 adult protocol. Based on data from UKALL XII, a previous UKALL protocol that utilised native asparaginase [34], 47% adult patients were assumed to have received a transplant regardless of asparaginase regimen. It was assumed that all patients eligible for transplantation underwent transplantation post-induction, at which point their asparaginase treatment was ceased. Specific transplant costs and outcomes of transplantation were not accounted for in the analysis because the treatment regimen and associated patient outcome depend on transplant success or failure, when asparaginase is no longer used.

### Quality of life

In the absence of UK-specific health-related quality of life data for ALL, the relative differences between population norms and the ALL treatment phases were applied to published UK EQ-5D population norms [37], adjusting for baseline patient age (Table 1). Utilities across treatment phases were reported by Furlong et al., 2012 [28] using the Health Utilities Index (HUI2 and HUI3).

QALYs were accrued by applying the age-adjusted utility values from UK (England) population norms to the proportion of patients in each health state over time (Markov traces). Relative utility decrements were applied for the treatment phases from English age-adjusted population norms.

A utility decrement of 0.014 was applied to reflect the impact of hypersensitivity on patient health-related quality of life [31]. Patients who were event-free at 5 years were considered to be cured and in the lifetime extrapolation they were given the same utility as the general population. Patients who were in the R/ST state could not transition to the EFS state and were given a decreased utility of 20%.

### Cost inputs

Cost parameter inputs to the model included unit drug costs, administration costs and adverse event management costs. Transplantation costs and outcomes were not accounted for, as these patients no longer receive asparaginase. The age-specific patient body surface area was derived from a weighted average (57% males and 43% females) of the DuBois and Mosteller formulas [38] computed from UK growth charts up to the age of 18 years. Beyond 18 years of age, a body surface area of 1.79 m^2^ was assumed, based on the UK average [33]. Administration cost and adverse event cost per hypersensitivity occurrence were assumed to be the same for all asparaginases.

### Scenario and sensitivity analyses

Uncertainty was assessed using scenario and sensitivity analyses. Scenario analyses were conducted to test the impact on the results of several plausible clinical scenarios, such as assuming a 100% paediatric population or 100% adult population. Deterministic sensitivity analyses were performed on all inputs included in the model apart from the dosing and treatment regimens. For both the paediatric and adult model, a range of parameters (risk distribution, utility decrements, risks and impact of hypersensitivity, 5-year outcomes, impact of R/ST, drug and administration costs) were considered for the probabilistic sensitivity analysis to investigate their collective impact on the ICER based on their known standard error (SE) around the base case estimate. A standard error of 5% was assumed where the SE was unknown.

## Results

### Base case incremental cost effectiveness analysis results

The base case results are presented in Table 2. The current therapy strategy (pegaspargase used first line followed by Erwinia asparaginase) was shown to dominate the old therapy strategy (first-line native asparaginase followed by Erwinia asparaginase), resulting in a reduction in overall cost of £4741 and an increase in QALYs of 0.0504.

**Table 2:** Base case cost-effectiveness results^a^

Strategy	Total		Incremental^b^		ICER (£)
	Cost (£)	QALYs	Cost (£)	QALYs	
Current therapy	£7871	17.3431	–	–	–
Old therapy	£12,612	17.2926	£4741	−0.0504	Dominated^c^

Current therapy strategy: pegaspargase followed by Erwinia asparaginase in cases of hypersensitivity

Old therapy strategy: native asparaginase followed by Erwinia asparaginase in cases of hypersensitivity

*QALY* quality-adjusted life years

^a^This table has previously been published in an adapted format [39–41]

^b^Incremental Cost or Incremental QALYs compared with the current therapy strategy

^c^Dominated indicates that the current therapy strategy resulted lower costs and more QALY gain versus the old therapy strategy

Disaggregated results of the cost components across all treatment strategies are presented in Additional file 2: Table S1. The current therapy strategy reduced overall costs compared with the old therapy strategy and administration costs were the major driver for cost reduction (82.1% of the overall cost reduction).

### Probabilistic sensitivity analysis

**Fig. 2:**
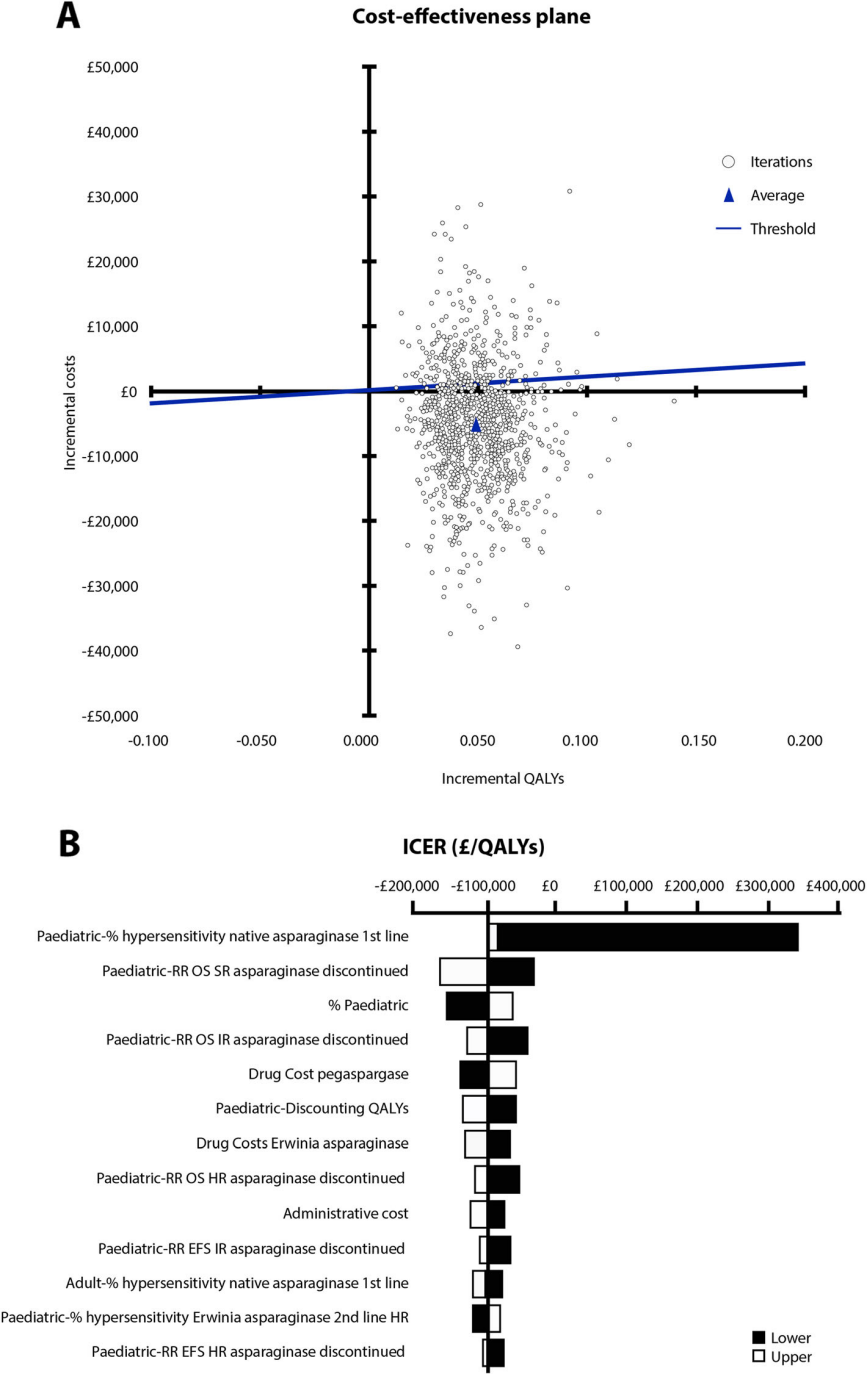
**a** Cost-effectiveness plane for the current therapy strategy versus the old therapy strategy. The results of 1000 simulations were plotted on the cost-effectiveness plane, with the majority of the simulations and probabilistic mean falling in the southeast quadrant. This indicated that the current therapy strategy was the dominant treatment strategy. Current therapy strategy: pegaspargase followed by Erwinia asparaginase. Old therapy strategy: native asparaginase followed by Erwinia asparaginase. QALY, quality-adjusted life years. **b** Tornado plot for incremental cost-effectiveness ratio of the current therapy strategy versus the old therapy strategy. Deterministic sensitivity analysis results indicated that ICER was stable for most parameters. The hypersensitivity rate for first-line treatment with native asparaginase for the paediatric population had the greatest impact on the ICER. Current therapy strategy: pegaspargase followed by Erwinia asparaginase. Old therapy strategy: native asparaginase followed by Erwinia asparaginase. EFS, event-free survival; HR, high risk; ICER, incremental cost-effectiveness ratio; IR, intermediate risk; OS, overall survival; QALY, quality adjusted life years; RR, relative risk; SR, standard risk

The results of 1000 simulations were plotted on the cost-effectiveness plane (Fig. 2a). All simulation results landed in the north-east and south-east quadrants of the cost-effectiveness plane, indicating that the current therapy strategy was always more effective than the old therapy strategy. Furthermore, the majority of the simulations and the probabilistic mean fell in the south-east quadrant, indicating that the current therapy strategy was a dominant (less costly and more effective) treatment strategy. When compared with the old therapy strategy, the current therapy strategy had a 77.9% probability of being below the £20,000 willingness-to-pay threshold.

### Deterministic sensitivity analysis

A tornado diagram was generated from the deterministic sensitivity analysis of all inputs included in the model, apart from the dosing and treatment regimens (Fig. 2b). Figure 2b shows the 13 parameters for which the ICER was most sensitive and indicates that the ICER was stable for the variation in most parameters. The ICER was unstable, however, when the hypersensitivity rate for first-line treatment with native asparaginase was varied in the paediatric and AYA population. Although clinically unlikely, when the hypersensitivity rate was set to 0%, or no risk of hypersensitivity was assumed for first-line native asparaginase, the ICER reached £340,630/QALY.

### Scenario analysis

A range of scenarios were conducted to explore the impact of uncertainty in model parameters. The ICER for the current therapy strategy versus the old therapy strategy for each scenario is presented in Additional file 2: Table S2. Pegaspargase remains cost-effective versus all treatment strategies for all scenarios, including the SmPC-approved pegaspargase dose of 2500 IU/m^2^ [13]. In the scenarios tested, the current therapy strategy was dominant compared with the old therapy strategy (Additional file 2: Table S2).

## Discussion

The UK-based cost-utility analysis presented in this paper shows that pegaspargase used as a first-line treatment is more effective and less costly than first-line native asparaginase. The model reflects the current standard of care with ALL in the UK for paediatric, AYA and adult patients (up to 65 years). The favourable hypersensitivity rates with first-line pegaspargase was a key driver in the model contributing to considerable cost savings compared with first-line native asparaginase, with additional reduction in administration cost due to fewer injections [13, 15, 16]. Cost savings based on lower hypersensitivity and fewer administrations of pegaspargase were similarly found by Tong and colleagues in the context of the ALL-10 MR protocol in the Netherlands [21] and earlier studies reported a similar or favourable overall cost for pegaspargase compared with native asparaginase [22, 23].

Although there is no comparative effectiveness evidence available for pegaspargase at a dose of 1000 IU/m^2^ versus 2000–2500 IU/m^2^, as recommended in the SmPC, it was considered appropriate to take the lower dose of pegaspargase into consideration because this is reflective of the dose considered as the standard of care in UK clinical practice [25]. The 1000 IU/m^2^ dose is also used in the Nordic NOPHO ALL 2008 protocol [42] and was one of the doses evaluated in the German GMALL 07/2003 protocol [43], while a 1500 IU/m^2^ dose of pegaspargase is used in the Dutch DCOG ALL-11 protocol [44]. It should be noted that although a lower dose was modelled versus the SmPC-recommended doses, each administration was considered to be equal to one 3750 IU vial of pegaspargase because unused product should be discarded [3]. In addition, it is standard clinical practice to cap the pegaspargase dose at one 3750 IU vial in protocols where a 2000 or 2500 IU/m^2^ dose is used [45–47]. Therefore the calculated cost of pegaspargase would be equivalent, regardless of the dose modelled. In addition, the scenario analysis also assessed the 2500 IU/m^2^ pegaspargase dose, recommended for patients ≤21 years old [13], and reached the same conclusion. The NICE Technology Appraisal Committee reviewed the model as part of the assessment for reimbursement of pegaspargase for England & Wales and concluded that it could recommend pegaspargase as a cost-effective use of NHS resources for treating ALL in children, young people and adults with untreated, newly diagnosed disease [25].

It has been suggested that the budget perspective of agencies such as NICE is relatively narrow and that a wider societal perspective on value should be considered [48]. Others argue that both perspectives are necessary, depending on the aim of the analysis, and that a strict healthcare perspective cost-effectiveness ratio is relevant when policy makers are considering health care goals [49]. Taking a societal perspective, all significant health outcomes and costs experienced by everyone affected by the medical intervention should be included [49], which includes a huge number of factors, not all of which can be addressed here. Although not modelled, switching from administration once every 2 weeks with pegaspargase [13] to three times per week with Erwinia asparaginase [16] would lead to increased transport costs for all patients and could result in productivity loss for patients or parents/carers during treatment stages following induction when treatment as an outpatient may be possible [50]. These costs and productivity losses would have to be considered from the first-line setting with the old treatment strategy, as native asparaginase is administered three times per week [15]. Therefore, accounting for these additional factors from a societal perspective would further favour the current versus the old treatment strategy, suggesting that our findings may be conservative. A recent study in The Netherlands on the cost-effectiveness of switching to Erwinia asparaginase versus discontinuing asparaginase following a hypersensitivity reaction to pegaspargase evaluated cost per life years saved using a wider perspective, including hospital costs, burden of switching and patient experience of the hypersensitivity reactions [51]. They found that although the cost was increased by switching to Erwinia asparaginase, the long-term clinical benefit and reduction in relapse resulting from this intervention versus discontinuing asparaginase completely merited their recommendation of the switch. Although using different scenarios to ours, their wider perspective still found in favour of first-line pegaspargase followed by a switch to Erwinia asparaginase, where necessary.

Limitations to the current analysis are primarily the lack of direct head-to-head data, especially in the adult population. Key inputs were validated by experts to ensure the values used were reflective of UK experience, especially as protocols differ between countries, so the applicability of these data in other circumstances should be considered with caution. The assumption was made that the current therapy strategy and the old therapy strategy were equivalent in terms of OS and EFS. Although this assumption was endorsed by clinical experts in the UK, it requires further validation with clinical data. Additionally, the findings of our model may not be applicable to patients over 65 years of age, as these patients were excluded in the UKALL14 protocol [3, 7].

Our deterministic sensitivity analysis indicated a stable ICER across most parameters, however, it was unstable when the hypersensitivity rate was varied for first-line treatment with native asparaginase in paediatric patients and, in particular, when the value was set at 2% or below, which in reality would be highly unlikely. ICER instability has been discussed as a limitation in a proposal of reporting standards, which recommends that average cost-effectiveness ratios be reported in parallel to allow better interpretation of cost-effective analysis data [52]. The ICER data should therefore be interpreted within this context and in conjunction with the other analyses performed.

The generalisability of results from this model may be limited where treatment practices, healthcare systems and/or cost structures are significantly different from the UK. While mapping results to other countries should be done with caution, it is clear that several fundamental attributes of pegaspargase treatment are pivotal in driving the cost-effectiveness results demonstrated by this model, despite its development specifically for clinical practice in the UK. Pegaspargase has a longer half-life and slower clearance than the non-pegylated forms of asparaginase, and thus can be given less frequently to patients [10, 53–55]. Furthermore, as native asparaginase is only available in an injectable form and intramuscular injections may be painful, less frequent injections of pegaspargase are preferable [56, 57]. Additional analyses will be required in countries that do not routinely include Erwinia asparaginase as the second-line asparaginase within the care pathway for ALL patients, due to cost or availability considerations. In response to this, two recent single-centre studies in the US reported that the implementation of policies, including staff training, therapeutic drug monitoring and premedication, significantly reduced the incidence of hypersensitivity reactions with pegaspargase and the need to switch to Erwinia asparaginase [12, 24], thereby resulting in substantial cost savings and clinical benefits for patients. These encouraging data, along with our findings that low hypersensitivity rates with pegaspargase substantially contributed to the cost effectiveness of the current treatment strategy, suggest that the first-line use of pegaspargase could be the key to cost-effective asparaginase treatment of ALL.

## Conclusions

Allowing for the stated assumptions and limitations, this analysis demonstrates that pegaspargase, as part of multi-drug chemotherapy, is a cost-effective option for the treatment of newly diagnosed ALL in children, young adults, and adults.

## Supplementary information

**Additional file 1.** CHEERS checklist.

**Additional file 2: Table S1.** Cost Analysis by category. **Table S2.** Scenario Analyses.

## Data Availability

The data, models, and methodology used in the research are proprietary. Associated NICE guidance documents can be found at https://www.nice.org.uk/guidance/ta408

## Abbreviations

ALL: Acute lymphoblastic leukaemia
DT: Decision tree
EFS: Event-free survival
HR: High-risk
ICER: Incremental cost-effectiveness ratio
IR: Intermediate-risk
ISPOR: International Society for Pharmacoeconomics and Outcomes Research
ISPOR-SMDM: International Society for Pharmacoeconomics and Outcomes Research and Society for Medical Decision Making
NHS: National Health Service
NICE: National Institute for Health and Care Excellence
OS: Overall survival
QALYS: Quality-adjusted life years
R/ST: Relapse/secondary tumour
SE: Standard error
SmPC: Summary of product characteristics
SR: Standard-risk
