# Supplementary material for: The cost-effectiveness of pegaspargase versus native asparaginase for first-line treatment of acute lymphoblastic leukaemia: a UK-based cost-utility analysis
Source: Health Econ Rev. 2019 Dec 29;9:40. doi: 10.1186/s13561-019-0257-3 (PMC6935472; doi:10.1186/s13561-019-0257-3)
Supplement: Supplementary file 1 — Additional file 1. CHEERS checklist. [file 13561_2019_257_MOESM1_ESM.docx]

| **Section/item Item** | **Item number** | **Recommendation** | **Reported on**  **page No**  **line No** |
| --- | --- | --- | --- |
| **Title and abstract** | | | |
| Title | 1 | Identify the study as an economic evaluation or use more specific terms such as “cost-effectiveness analysis”, and describe the interventions compared. | Title page  L1,2 |
| Abstract | 2 | Provide a structured summary of objectives, perspective, setting, methods (including study design and inputs), results (including base case and uncertainty analyses), and conclusions. | P1  L1–33 |
| **Introduction** | | | |
| Objectives | 3 | Provide an explicit statement of the broader context for the study.  Present the study question and its relevance for health policy or practice decisions. | P5  L80–84 |
| **Methods** | | | |
| Target population and subgroups | 4 | Describe characteristics of the base case population and subgroups analysed, including why they were chosen. | P4  L61–66  P6  L87–89  P7  L119–121  P8  L144–150 |
| Setting and location | 5 | State relevant aspects of the system(s) in which the decision(s) need(s) to be made. | P4  L61–72  P5  L78–80  P6  L87–89 & L102–107  P7  L119–121 |
| Study perspective | 6 | Describe the perspective of the study and relate this to the costs being evaluated. | P4  L76–80  P6  L102–107  P13  L255–262 |
| Comparators | 7 | Describe the interventions or strategies being compared and state why they were chosen. | P7  L19–130  Figure 1 |
| Time horizon | 8 | State the time horizon(s) over which costs and consequences are being evaluated and say why appropriate | P6  L96–100  P7  L115–116 |
| Discount rate outcomes and say why appropriate | 9 | Report the choice of discount rate(s) used for costs and | P6  L106–107 |
| Choice of health outcomes | 10 | Describe what outcomes were used as the measure(s) of benefit in the evaluation and their relevance for the type of analysis performed | P7  L109–112  P9  L156–164  P10  L180–187  P12  L243–246  P13  L249–252 |
| Measurement of  effectiveness | 11b | *Synthesis-based estimates:* Describe fully the methods used for identification of included studies and synthesis of clinical effectiveness data. | P6  L87–89  L105–106  P7  L109–112  L115–116  L119–121  P8  L136–138  L142–144  P9  L167–178  P10  L180–187  L190–194  L197–201  P11  L202–211  P12  L227–229 L237–246  P13  L248–249  L257–260  Table 1 |
| Measurement and valuation of preference based outcomes | 12 | If applicable, describe the population and methods used to elicit preferences for outcomes. | P11  L214–223  (UK protocol preference specific asparaginase doses) |
| Estimating resources and costs | 13b | *Model-based economic evaluation:* Describe approaches and data sources used to estimate resource use associated with model health states. Describe primary or secondary research methods for valuing each resource item in terms of its unit cost. Describe any adjustments made to approximate to opportunity costs. | P6  L87–89  L102–107  P7  L109–112  P12  L231–234  P13  L255–262  Supplementary Table 1 |
| Currency, price date, and conversion | 14 | Report the dates of the estimated resource quantities and unit costs. Describe methods for adjusting estimated unit costs to the year of reported costs if necessary. Describe methods for converting costs into a common currency base and the exchange rate | P6  L102–107 |
| Choice of model | 15 | Describe and give reasons for the specific type of decision analytical model used. Providing a figure to show model structure is strongly recommended | P6  L94–100  P7  L115–130  P8  L131–133  P9  L156–164  P13  L265–270  P14  L271–274  Figure 1 |
| Assumptions | 16 | Describe all structural or other assumptions underpinning the decision-analytical model. | P8  L136–138  P9  L159–162  L175–178  P10  L180–181  L185–187  L190–193  L197–199  P11  L202–204  L206–207  P12  L229–231  P13  L259–262  L265–267  P14  L273–274  Table 1 |
| Analytical methods | 17 | Describe all analytical methods supporting the evaluation. This could include methods for dealing with skewed, missing, or censored data; extrapolation methods; methods for pooling data; approaches to validate or make adjustments (such as half cycle corrections) to a model; and methods for handling population heterogeneity and uncertainty. | As for assumptions above and  P9  L163–164  P10  L186–187  P11  L207–211  P12  L237–246  P13  L265–270  P14  L271–274 |
| **Results** | | | |
| Study parameters | 18 | Report the values, ranges, references, and, if used, probability distributions for all parameters. Report reasons or sources for distributions used to represent uncertainty where appropriate. Providing a table to show the input values is strongly recommended. | P15–P16  L275–314  Table 1 |
| Incremental costs and outcomes | 19 | For each intervention, report mean values for the main categories of estimated costs and outcomes of interest, as well as mean differences between the comparator groups. If applicable, report incremental cost-effectiveness ratios. | Table 2  Supplementary Table 1 |
| Characterising uncertainty | 20b | *Model-based economic evaluation:* Describe the effects on the results of uncertainty for all input parameters, and uncertainty related to the structure of the model and assumptions. | P16  L308–314  Supplementary Table 2  Figure 2 |
| **Discussion** |  |  |  |
| Study findings, limitations, generalisability, and current knowledge | 22 | Summarise key study findings and describe how they support the conclusions reached. Discuss limitations and the generalisability of the findings and how the findings fit with current knowledge. | P17–20  L315–406 |
| **Other** |  |  |  |
| Source of funding | 23 | Describe how the study was funded and the role of the funder in the identification, design, conduct, and reporting of the analysis. Describe other non-monetary sources of support. | Title page document |
| Conflicts of interest | 24 | Describe any potential for conflict of interest of study contributors in accordance with journal policy. In the absence of a journal policy, we recommend authors comply with International Committee of Medical Journal Editors recommendations. | Title page document |
