# Supplementary material for: The cost-effectiveness of pegaspargase versus native asparaginase for first-line treatment of acute lymphoblastic leukaemia: a UK-based cost-utility analysis
Source: Health Econ Rev. 2019 Dec 29;9:40. doi: 10.1186/s13561-019-0257-3 (PMC6935472; doi:10.1186/s13561-019-0257-3)
Supplement: Supplementary file 2 — Additional file 2: Table S1. Cost Analysis by category. Table S2. Scenario Analyses. [file 13561_2019_257_MOESM2_ESM.docx]

**Table S1: Cost Analysis by category**

|  | **Average costs** | |
| --- | --- | --- |
| **Strategy** | **Current therapy** | **Old therapy** |
| Drug cost | £6,980 | £7,716 |
| Pegaspargase | £6,650 | £0 |
| Native asparaginase | £0 | £2,144 |
| Erwinia asparaginase | £330 | £5,571 |
| Administration cost | £878 | £4,769 |
| Pegaspargase | £839 | £0 |
| Native asparaginase | £0 | £4,145 |
| Erwinia asparaginase | £40 | £625 |
| Hypersensitivity | £12 | £127 |
| Total | £7,871 | £12,612 |
| **Absolute increment** | | |
| Drug cost | - | £735 |
| Administration cost | - | £3,891 |
| Hypersensitivity | - | £115 |
| Total | - | £4,741 |
| **Percent absolute increment** | | |
| Drug cost | - | 15.5% |
| Administration cost | - | 82.1% |
| Hypersensitivity | - | 2.4% |
| Total | - | 100% |

Current therapy strategy: pegaspargase followed by Erwinia asparaginase

Old therapy strategy: native asparaginase followed by Erwinia asparaginase

**Table S2: Scenario Analyses**

| **Scenario** | **Current therapy strategy vs old therapy strategy** |
| --- | --- |
| **Base case** | P |
| **100% paediatric population** | P |
| **100% adult population** | P |
| **Minimum cost of hypersensitivity** | P |
| **Maximum cost of hypersensitivity** | P |
| **1.5% discount rate for paediatric population** | P |
| **Pegaspargase dose as per SmPC** | P |
| **Minimum cost of native asparaginase** | P |
| **Maximum cost of native asparaginase** | P |
| **Average paediatric age = 1** | P |
| **Average paediatric age = 18** | P |

P = pegaspargase dominant
Current therapy strategy: pegaspargase followed by Erwinia asparaginase

Old therapy strategy: native asparaginase followed by Erwinia asparaginase

SmPC, summary of product characteristics
